# Impact of transition to a subterranean lifestyle on morphological disparity and integration in talpid moles (Mammalia, Talpidae)

**DOI:** 10.1186/s12862-019-1506-0

**Published:** 2019-09-12

**Authors:** Gabriele Sansalone, Paolo Colangelo, Anna Loy, Pasquale Raia, Stephen Wroe, Paolo Piras

**Affiliations:** 1 Form, Evolution and Anatomy Research Laboratory, Zoology, School of Environmental and Rural Sciences, University of New England, Armidale, NSW 2351 Australia; 2 Department of Sciences, Roma Tre University, Largo San Leonardo Murialdo 1, I-00146 Rome, Italy; 3 National Research Council, Institute of Research on Terrestrial Ecosystems, Via Salaria km 29.300, 00015 Monterotondo (Rome), Italy; 4 Environmetrics Lab, Dipartimento STAT, Università del Molise, I-86090 Pesche, Italy; 5 Università degli Studi di Napoli Federico II, Department of Earth Sciences, Environment and Resources, L.go San Marcellino 10, 80138 Naples, Italy; 6 Dipartimento di Scienze Cardiovascolari,Respiratorie, Nefrologiche, Anestesiologiche e Geriatriche, “Sapienza”, Università di Roma, Via del Policlinico 155, 00161 Rome, Italy; 7 Dipartimento di Ingegneria Strutturale e Geotecnica, Sapienza, Università di Roma, Via Eudossiana 18, 00100 Rome, Italy

**Keywords:** Geometric morphometrics, Disparity, Integration, Humerus, Mandible, Phylogenetic comparative methods, Partial warps

## Abstract

**Background:**

Understanding the mechanisms promoting or constraining morphological diversification within clades is a central topic in evolutionary biology. Ecological transitions are of particular interest because of their influence upon the selective forces and factors involved in phenotypic evolution. Here we focused on the humerus and mandibles of talpid moles to test whether the transition to the subterranean lifestyle impacted morphological disparity and phenotypic traits covariation between these two structures.

**Results:**

Our results indicate non-subterranean species occupy a significantly larger portion of the talpid moles morphospace. However, there is no difference between subterranean and non-subterranean moles in terms of the strength and direction of phenotypic integration.

**Conclusions:**

Our study shows that the transition to a subterranean lifestyle significantly reduced morphological variability in talpid moles. However, this reduced disparity was not accompanied by changes in the pattern of traits covariation between the humerus and the mandible, suggesting the presence of strong phylogenetic conservatism within this pattern.

**Electronic supplementary material:**

The online version of this article (10.1186/s12862-019-1506-0) contains supplementary material, which is available to authorized users.

## Background

Understanding why some clades achieve a large morphological, behavioral and ecological diversity, while others do not, represents a central aim in evolutionary biology. Studies addressing this question usually try to identify the factors allowing high phenotypic diversity, or constraining its realization [1]. Key innovations and the transition to novel ecological niches are generally thought to promote morphological variation of clades, but might also reduce taxonomic diversification via niche specialization and stabilizing selection [1, 2]. These differential effects of trait acquisition or ecological transition on phenotypic diversity between clades are best exemplified by sister clades, where one clade shows higher phenotypic diversity than the others [3, 4]. In this context, it has been proposed that phenotypic traits covariation and modularity could promote differences in disparity among clades [5]. Simulation studies have shown that traits covariation may drive morphological variability along different axes of variation, resulting in either less or more disparity depending on the relationship between selection pressures and the major axes of variation [1, 5]. Ecological transitions are of particular interest because of their large influence on phenotypic diversification [3, 4, 6]. Specifically, while species shifting into new niches are likely to evolve adaptations allowing them to exploit the new ecological settings, those remaining within the ancestral niche should retain plesiomorphic anatomical structures [7, 8]. Textbook examples of this are found among archosaurs [9], anuran and caecilian amphibians [4, 10], and primates [11], among others.

A notable ecological transition is represented by the colonization of the subterranean ecotope by different, unrelated taxa (rodents, insectivores and marsupials [12, 13]). Different studies on rodents have demonstrated how the colonization of the subterranean ecotope could dramatically influence species morphology and how digging specialization shapes the evolution of forelimbs and the cranio-dental complex [14–16]. One of the most spectacular examples of adaptation to subterranean life is represented by moles. The mammalian family Talpidae includes ambulatorial (Uropsilini), semi-aquatic (Desmanini and Codylurini), semi-fossorial (Urotrichini and Neurotrichini) and fully subterranean species (Scalopini and Talpini). The colonization of such a wide array of environments was realized by the combination of different behavioral, physiological and morphological adaptations [17–21] especially evident in the forelimbs of digging species [17, 18, 22, 23]. Such wide array of morphological adaptations makes talpids an ideal group for investigating the phenotypic effects of a major ecological transition, such as the colonization of the subterranean habitat.

In the present study, we provide a comprehensive morphological analysis of the humerus and mandible of all extant talpid genera. The humerus is widely known to be a good proxy for locomotor behavior in moles [19, 20, 23], while the mandible is obviously linked to feeding [24–27]. We used a 2D geometric morphometrics to quantitatively assess shape variation in both humeri and mandibles between subterranean and non-subterranean moles. Then, we investigated different aspects of these bones’ shape covariation under a phylogenetically-informed scenario. Specifically, we compared the strength and direction of phenotypic covariation between the two anatomical structures and between subterranean and non-subterranean moles. The need for coordination and integration between the feeding and locomotor apparatuses has been shown for different vertebrate taxa [28, 29]. In particular, highly specialized behaviors may require a more complex interaction between different parts of an organism, resulting in increased covariation [30]. We hypothesized that the species adapted to the subterranean lifestyle might display stronger trait covariation between the humerus and the mandible due to the functional constraints imposed by highly demanding digging kinematics and by the limited range of food items available underground [12, 17]. We further determined if the potential differences in morphological disparity between the species falling in the two ecotopes could be ascribed to variation in levels of phenotypic covariation.

## Methods

### Material

We examined left 365 mandibles and 463 left humeri belonging to adult individuals of 37 extant talpid species. Specimens are stored in the following institutions: ISEZ-PAN, Krakow, Poland, Tsukuba Natural History Museum, Tsukuba, Ibaraki, Japan; Museu de Historia Natural, Lisboa, Portugal; Natural History Museum, London, UK; BSPG, Munich, Germany; Wien Natural History Museum, Wien, Austria; LACM, Los Angeles, USA; UCMP, Berkeley, USA; “La Specola” Museo di Storia Naturale di Firenze, Italy and Museo di Anatomia Comparata G.B. Grassi, “Sapienza” Università di Roma, Rome, Italy. Details about the sample are summarized in Table 1. We separated the species under investigation into two groups: non-subterranean and subterranean. Following [12, 13, 31], subterranean species spend most of their life underground and come above the ground only incidentally, whereas non-subterranean species lack the extreme underground specializations and spend a considerable amount of time above the ground (e.g. foraging). Groupings at the species level are reported in Table 1 (Further details on the sampling effort can be found in Additional file 1). Clade assignation follows [32].

**Table 1:** Species, sample size, relative lifestyle and clade assignation

Species	n Humerus	n Mandible	Lifestyle	Clade
*Uropsilus andersoni*	3	6	Non-subterranean	Uropsilini
*U. gracilis*	3	2	Non-subterranean	Uropsilini
*U. investigator*	4	5	Non-subterranean	Uropsilini
*U. soricipes*	5	4	Non-subterranean	Uropsilini
*Desmana moschata*	7	11	Non-subterranean	Desmanini
*Galemys pyrenaicus*	8	12	Non-subterranean	Desmanini
*Dymecodon pilirostris*	8	12	Non-subterranean	Urotrichini
*Urotrichus talpoides*	12	9	Non-subterranean	Urotrichini
*Neurotrichus gibbsii*	17	12	Non-subterranean	Neurotrichini
*Scaptonyx fusicaudus*	2	4	Non-subterranean	Neurotrichini
*Condylura cristata*	6	11	Subterranean	Condylurini
*Euroscaptor klossi*	2	4	Subterranean	Talpini
*E. longirostris*	4	3	Subterranean	Talpini
*E. malayana*	3	6	Subterranean	Talpini
*E. micrura*	2	11	Subterranean	Talpini
*E. mizura*	4	10	Subterranean	Talpini
*Mogera imaizumii*	17	18	Subterranean	Talpini
*M. insularis*	7	12	Subterranean	Talpini
*M. kanoana*	10	9	Subterranean	Talpini
*M. tokudae*	24	20	Subterranean	Talpini
*M. wogura*	29	30	Subterranean	Talpini
*Parascaptor leucura*	4	13	Subterranean	Talpini
*Scaptochirus moschatus*	12	5	Subterranean	Talpini
*Talpa altaica*	15	9	Subterranean	Talpini
*T. caeca*	21	7	Subterranean	Talpini
*T. caucasica*	7	5	Subterranean	Talpini
*T. europaea*	31	8	Subterranean	Talpini
*T. levantis*	9	6	Subterranean	Talpini
*T. occidentalis*	46	12	Subterranean	Talpini
*T. romana*	59	25	Subterranean	Talpini
*T. stankovici*	11	4	Subterranean	Talpini
*Parascalops breweri*	4	6	Subterranean	Scalopini
*Scalopus acquaticus*	23	20	Subterranean	Scalopini
*Scapanulus oweni*	2	2	Subterranean	Scalopini
*Scapanus latimanus*	17	8	Subterranean	Scalopini
*Sc. orarius*	6	9	Subterranean	Scalopini
*Sc. townsendii*	19	15	Subterranean	Scalopini

### Phylogenetic tree

**Fig. 1:**
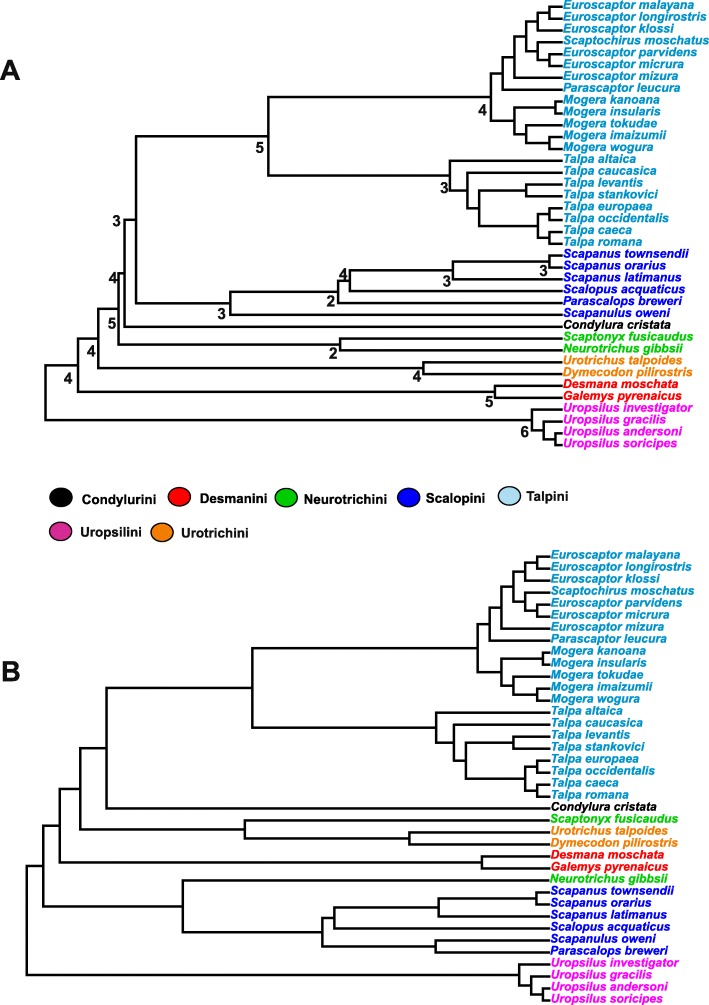
**a.** The phylogenetic hypothesis based on morphological characters. Values at nodes are Bremer decay indices. **b.** The topology based on molecular data

The phylogenetic history of Talpidae is highly debated. Despite a growing number of papers on the subject matter, there is still a lack of agreement between the different phylogenetic hypotheses [18, 21, 33–40]. In particular, morphological and molecular approaches conflict upon the position of Scalopini, the monophyly of Urotrichini and the position of *Condylura* [20, 32, 38–41]. Since a systematic revision of Talpidae phylogeny is beyond the scope of the present work, we decided to use two different phylogenetic hypotheses when using phylogenetic comparative methods. The first is based on molecular data, where Neurotrichini (*Scaptonyx* and *Neurotrichus*) are a polyphyletic group [40]. However, because this phylogenetic tree does not include all the species under investigation in the present paper, we built a synthetic phylogeny (an informal supertree using the Mesquite software [42]) supplemented with additional data to resolve the relationships within the genus *Talpa* [39] and within the genus *Euroscaptor* [41]. The second phylogenetic hypothesis is based on trees produced by maximum parsimony cladistic analysis of morphological characters based on a published character matrix we developed elsewhere [32]. The character matrices were analysed using PAUP 4.0 a147 [43, 44] using a heuristic search and stepwise addition, with a random addition sequence of 1000 replicates. The phylogenetic comparative analyses (see below) were applied on a strict consensus tree computed on the three most parsimonious trees found. The character matrix and relative character list are presented in Additional file 2. The time calibration for branches has been derived from a thorough review of the palaeontological literature on the subject matter [20, 31, 39]. For the time calibration we considered: the ages of the first occurrence of extant species and the molecular clock estimate (when available). The time calibration has been performed using the Stratigraphic Tool in the Mesquite software [42]. The two phylogenies are presented in Fig. 1, further details on the cladistics analysis are presented in Additional file 2, whereas detailed information about the time calibration are presented in Additional file 3.

### Geometric morphometrics and shape analysis

**Fig. 2:**
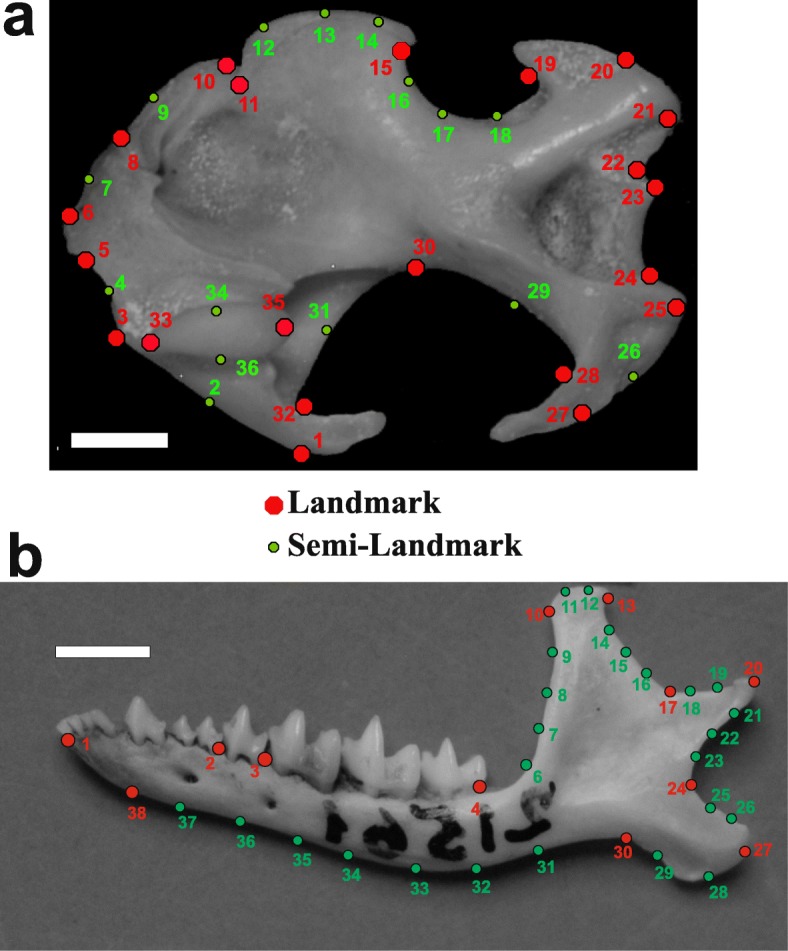
**a.** Landmarks and semi-landmarks digitized on the humerus in caudal norm: 1) lateral end of greater tuberosity; 2) articular facet of clavicula; 3) proximal edge of the articular facet of clavicula; 4) bicipital notch; 5) proximal end of lesser tuberosity; 6) medial edge of minor tuberosity; 7) lateral edge of lesser tuberosity; 8) bicipital ridge; 9) middle point of bicipital tunnel; 10) lateral end of scalopine ridge; 11) proximal end of teres tubercle; 12–14) surface of teres tubercle; 15) distal end of teres tubercle; 16–18) minor sulcus; 19) posterior margin of lateral epicondyle; 21–22) lateral epicondyle; 22–24) trochlear area; 25–27) medial epicondyle; 28) posterior margin of medial epicondyle; 29–32) greater sulcus; 33–36) humeral head. Scale bar is 1 mm. **b.** Landmarks and semi-landmarks digitized on the mandible. 1) Anterior tip; 2) anterior end of p4; 3) anterior end of m1; 4) posterior end of m3; 5–9) anterior profile of the coronoid process; 10–13) profile of the condyle of coronoid process; 14–17) posterior profile of the coronoid process; 18–24) condylar process; 25–30) profile of the angular process; 31–38) profile of the orizontal ramus. Scale bar is 1 mm

Humeri and mandibles were photographed in caudal view at a fixed distance of 50 cm using a Nikon D100 camera with a Micro-Nikkor 105-mm lens. We digitized 22 landmarks and 14 semi-landmarks on the humerus and 12 landmarks and 26 semi-landmarks on the mandible (Fig. 2a and b) using the tpsDig2 software [45]. The humeral and mandibular landmark configurations were derived from [40, 46–48] respectively. Semi-landmarks were used to capture the morphology of complex outlines where homologous anatomical points were missing. Semi-landmarks assume that curves or contours are homologous among specimens [49]. Successively, a generalized Procrustes analysis (GPA) [50], implemented with the procSym() function in the R-package “Morpho” [51], was used to rotate, translate, and scale landmark configurations to unit centroid size (CS = the square root of the sum of squared distances of the landmarks from their centroid [52]). To visualize the multivariate ordination of the aligned specimens, we performed a between-group PCA (bgPCA), using the function groupPCA() included in the R-package “Morpho”, considering the species as groups. The bgPCA provides a projection of the data onto the principal components of the group means, resulting in an ordination of the shape variables between the group means. The new axes are orthogonal and can be computed even when the per-group data matrices are not of full rank. This method provides a good performance when the number of observations in each group is smaller than the number of variables [53, 54].

Because we have a different number of specimens for humeri and mandibles for each species, all of the following analyses were performed on per-species averaged data. The significance of the observed shape changes between subterranean and non-subterranean species was evaluated by performing a Procrustes ANOVA on aligned Procrustes coordinates using the function procD.lm() included in the R package “geomorph” [55]. To measure shape disparity among non-subterranean and subterranean species we used Procrustes variance, which is the sum of the diagonal elements of the group covariance matrix divided by the number of observations in the group using the function morphol.disparity() from the R package “geomorph” [56, 57]. In order to visualize shape changes in ordination plots we chose to use the method described in [57]. There it was suggested that a useful way to visualize local, infinitesimal variation within a deformation grid is to use the Jacobian (*J*) of the Thin Plate Spline interpolation function. *J* captures very local information as localized variation in the non-affine component of the deformation using derivatives of the used interpolation function (TPS in our case). In 2D *J* is a 2 × 2 matrix that can be evaluated at any point within a body. The logarithm of its determinant represents the change in the area in the region about the interpolation point. Values < 0 indicate that, with respect to the source (here the sample consensus), the target (here the PC’s extremes) experiences a reduction in the local area, while values > 0 indicate an enlargement.

### Evolutionary allometry and size correction

Multivariate regressions between shape and size data were applied to determine the presence of evolutionary allometry in both the humerus and the mandible. To test for differences in slope among subterranean and non-subterranean species, we ran a permutational multivariate analysis of covariance, using Procrustes coordinates as dependent variables, centroid size (CS) as an independent variable and the two groups as factor [58, 59]. To test the effect of size on morphological disparity the CS was included in the model as a covariate. In order to test the potential effect of size on the pattern of morphological covariation we repeated each of the analyses described below after computing size-free Procrustes coordinates.

### Measurement error

The measurement error associated with the digitization of landmarks was measured on three replicates of 100 specimens for each dataset (humeri plus mandibles). The mean Procrustes distances between all the combinations of pairs of specimens were computed for each replicated dataset using the TPSsmall software [60]. We calculated the mean Procrustes distances for each triplet of the same subjects occurring in the three replicas. We then computed the averages of all the mean values of the minimum and maximum values of each triplet. The amount of digitization error, with respect to the total variation in the shape, can be expressed as a percentage. We calculated the ratio of the mean value for total digitization and the mean of the total dataset.

### Trait covariation, strength and direction

It has been recently noted [61] that sliding semi-landmarks using the minimum bending energy (BEN) approach may result in increased covariation between modules. Because we used semi-landmarks in our dataset, we repeated all the following integration analyses using shape coordinates derived using both the minimum BEN and minimum Procrustes distances (PRD) approaches in order to evaluate any potential discrepancy in the results. We report here that we did not find any significant discrepancy when using either sliding methods, hence we present only the results obtained from the analyses performed on the shape coordinates derived after using the minimum BEN approach.

We assessed the covariation between the humerus and the mandible using partial least squares (PLS) analyses [52, 62]. PLS is suitable for the study of covariation between two sets of variables in several groups. We quantified the covariation for each pair of axes by means of a correlation coefficient, whose significance is addressed by means of permutation under the null hypothesis that the distribution of specimens on one axis has no bearing on the distribution on the other axis [63]. Adams and Collyer [64] proposed a new strategy to compare the strength of PLS focusing on the first singular vector pair. The authors proposed a standardized test statistic (a z-score) for measuring the degree of morphological integration between sets of variables. The z-scores can be used to test for differences (via ANOVA) among groups. We used the compare.pls() function from the R package “geomorph” to compare the effect sizes, measured as standard deviates, z, and performs two-sample z-tests, using the pooled standard error from the sampling distributions of the PLS analyses. This tests for differences in the strength of covariation, whereas nothing is known about its direction. The orientation of integration patterns in space can be interpreted as the rate of shape changes in one module relative to the rate of shape changes in the other. This aspect is very important as it may reveal whether a common pattern of shape changes within modules exists between clades. In fact, groups may show similar integration coefficients but have different integration patterns [65, 66]. In order to investigate this issue, we performed separate major axis (MA) analyses on the different clades shapes on the space identified by the first pair of PLS axes [65, 66]. MA is particularly suitable here because of its ‘symmetry,’ i.e., residuals are computed orthogonally to the line of best fit and this is coherent with PLS aims. It does not require the classic assumption of dependence-independence relationship [65]. MA slopes were then compared through pairwise ANOVA, using lifestyle categories as groups.

### Global integration

Recently, Bookstein [67] proposed a new method to evaluate morphological integration “intrinsically” to a structure. This method tests the null hypothesis of “self-similarity” (e.g., the absence of any interpretable change at any spatial scale) in a collection of shapes and it is based on a linear regression of log partial warps variance against their proper log bending energy (i.e., the log of eigenvalues of the bending energy matrix computed on the consensus). Here, a regression slope less than − 1 indicates “integration” whereas a slope greater than − 1 indicates “dis-integration”. If the regression slope is exactly − 1 data can be considered “self-similar” (for further details refer to [67] and to Additional file 2). Finally, we compared the resulting slopes between subterranean and non-subterranean species using the R package RRPP [68].

### Phylogenetic non-independence and phylogenetic signal

The phylogenetic signal was calculated for the shape data using the K_mult_ statistic, a method that measures the similarity of trait values in relation to a Brownian motion model of evolution. It is specifically designed to address the challenge of working with high-dimensional landmark configurations [69].

The significance of the observed shape changes between subterranean and non-subterranean species was evaluated by performing a Procrustes ANOVA in a phylogenetic framework on aligned Procrustes coordinates using the function procD.pgls() included in the R package “geomorph” [49, 55]. We quantified the degree of phylogenetic morphological integration between the humerus and the mandible using partial least squares (PLS) analyses under the Brownian model of evolution using the function phylo.integration() from the geomorph R library [70].

## Results

### Measurement error

The digitization errors in the humeral and mandibular datasets were as low as 0.8 and 0.6% of the total variation, respectively. Because the measurement error was smaller than 5% in both datasets it could be safely assumed its effect on the results was negligible [39].

### Shape analysis

**Fig. 3:**
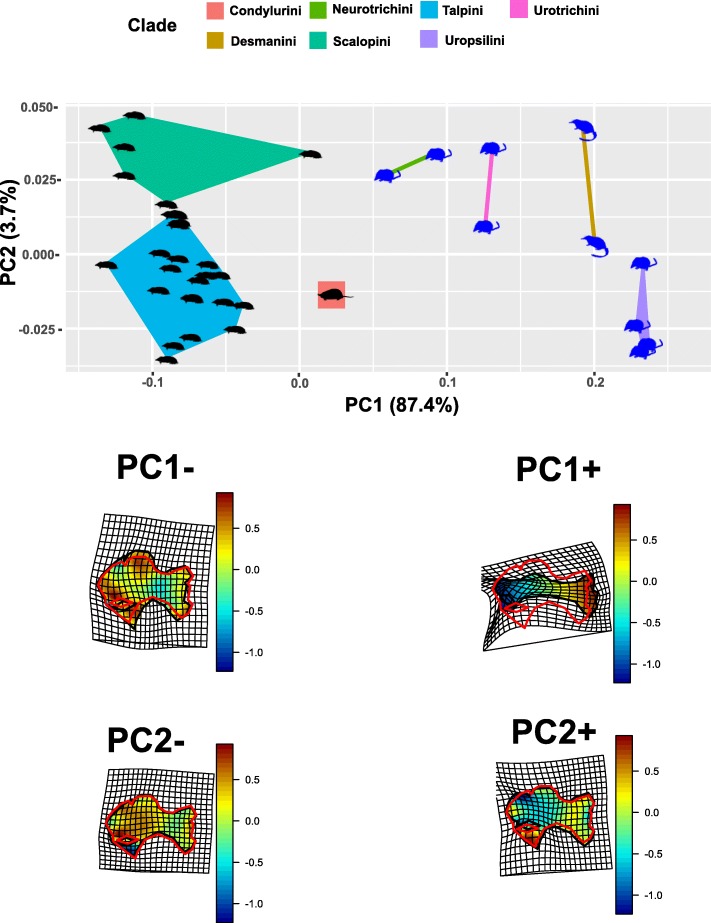
PC1/PC2 scatterplot of the bgPCA on humeral shape variables. Deformation grids refer to positive and negative extremes of the axes. Black silhouettes represent fossorial species, blue silhouettes represent non-fossorial species. Deformation grids shows color according to log(det(jacobian)) coming from the two-dimensional Thin Plate Spline first derivative evaluated within the body. Values < 0 indicate that, with respect to the source (here the sample’ consensus), the target (here the PC’s extremes) experiences a reduction in the local area, while values > 0 indicate an enlargement. Animal silhouettes were available under Public Domain license at phylopic (http://phylopic.org/). Specifically, shrew-mole (http://phylopic.org/image/822c549b-b29b-47eb-9fe3-dc5bbb0abccb/), this image is available for reuse under the Public Domain Dedication 1.0 license; *Talpa europaea* (http://phylopic.org/image/0465d81c-0def-4478-af15-a075d472e957/), this image is available for reuse under the Public Domain Dedication 1.0 license; *Condylura* (http://phylopic.org/image/8b656a93-ecf3-4985-8f4d-1f5c032d1a27/), available for reuse and under the Creative Commons Attribution 3.0 Unported (https://creativecommons.org/licenses/by/3.0/) image by Didier Escouens (vectorized by T. Michael Keesey). Desmans (http://phylopic.org/image/f6146c1d-874f-45a8-9d5c-eff0d9df0802/), this image is available for reuse under the Public Domain Dedication 1.0

*Humerus*. The bgPCA plot shows that subterranean and non-subterranean moles are well separated in the morphospace (Fig. 3). Procrustes ANOVA performed on the shape variables returned highly significant difference (*p*-value =0.001). Along the PC1 (87.4% of the total variance) there is a clear distinction between the subterranean (negative values) and non-subterranean (positive values) species. The humerus, at negative values shows a robust configuration with a relevant enlargement of the proximal region, in particular of the pectoral crest and the teres tubercle. At positive values the humerus shows a slender configuration with an overall reduced proximal region. Along the PC2 (3.7% of the total variance) it is possible to separate the Talpini (from consensus to negative values) from the Scalopini (positive values). At negative values the humerus shows a wide and elongated pectoral crest, while at negative values the pectoral crest is rounded and short.

**Fig. 4:**
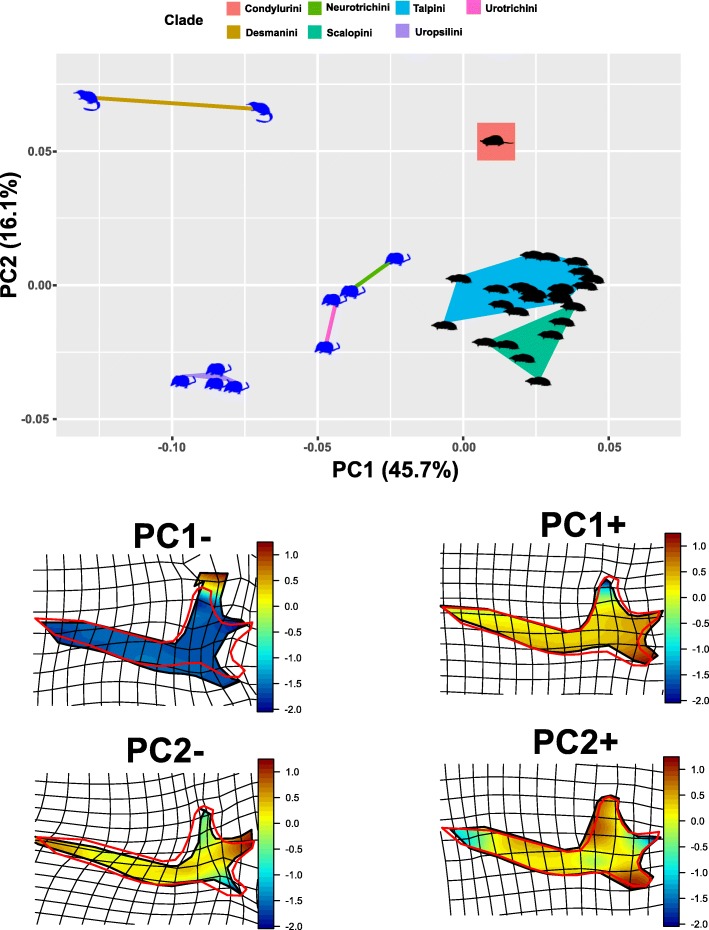
PC1/PC2 scatterplot of the bgPCA on mandibular shape variables. Deformation grids refer to positive and negative extremes of the axes. Black silhouettes represent fossorial species, blue silhouettes represent non-fossorial species. Deformation grids shows color according to log(det(jacobian)) coming from the two-dimensional Thin Plate Spline first derivative evaluated within the body. Values < 0 indicate that, with respect to the source (here the sample’ consensus), the target (here the PC’s extremes) experiences a reduction in the local area, while values > 0 indicate an enlargement. Animal silhouettes were available under Public Domain license at phylopic (http://phylopic.org/). Specifically, shrew-mole (http://phylopic.org/image/822c549b-b29b-47eb-9fe3-dc5bbb0abccb/), this image is available for reuse under the Public Domain Dedication 1.0 license; *Talpa europaea* (http://phylopic.org/image/0465d81c-0def-4478-af15-a075d472e957/), this image is available for reuse under the Public Domain Dedication 1.0 license; *Condylura* (http://phylopic.org/image/8b656a93-ecf3-4985-8f4d-1f5c032d1a27/), available for reuse and under the Creative Commons Attribution 3.0 Unported (https://creativecommons.org/licenses/by/3.0/) image by Didier Escouens (vectorized by T. Michael Keesey). Desmans (http://phylopic.org/image/f6146c1d-874f-45a8-9d5c-eff0d9df0802/), this image is available for reuse under the Public Domain Dedication 1.0

*Mandible*. The bgPCA plot showed that subterranean and non-subterranean moles are well separated in the morphospace (Fig. 4). Procrustes ANOVA performed on the shape variables returned a highly significant result (*p*-value = 0.001). Along the PC1 (45.7% of the total variance) there is a clear distinction between the subterranean (positive values) and non-subterranean (negative values) species. At negative values the mandible shows a straight and robust horizontal ramus, while the coronoid process is large and elongated, the condylar process is short and the angular process is pointed and slender. At positive values the mandible shows a bent horizontal ramus, while the coronoid process is slender and pointed, the condylar process is elongated and the angular process is robust and rounded. Along the PC2 (16.1% of the total variance) it is possible to separate the taxa showing a semi-aquatic lifestyle from all of the other moles. In particular, *Condylura* occupies the region of the morphospace marked by positive values, distinguished by a very slender and bent horizontal ramus, the coronoid and angular processes are extremely reduced, while the condylar process is robust and elongated. Desmans occupy the region of the morphospace marked by negative values, where the mandible shows a robust and straight horizontal ramus and an overall robust condylar region.

### Evolutionary allometry

The multivariate regressions revealed a significant interaction between shape and size for both the humerus and the mandible (F = 10.08, r^2^ = 0.22, *p*-value = 0.001; F = 14.25, r^2^ = 0.05, *p*-value = 0.001; respectively). Allometric trajectories were different between subterranean and non-subterranean taxa for both the humerus and the mandible (interaction *p*-value = 0.001, *p*-value = 0.003; respectively).

### Morphological disparity

Results from pairwise comparison of Procrustes variance are summarised in Table 2. For each structure (humerus and mandible) non-subterranean species always showed a significantly higher morphological disparity. Finally, the humerus proved to have an overall greater disparity when compared to the mandible (Procrustes variances: 0.018 and 0.0046, respectively). The inclusion of size (CS) as covariate did not impact the results from the previous analyses for both the humerus (*p*-value = 0.004) and the mandible (*p*-value = 0.001).

**Table 2:** Comparison of morphological disparity between subterranean and non-subterranean species. In bold are reported significant results

Morphological disparity	Procrustes variance		Procrustes variance no size	
	**subterranean/non-subterranean**	***p*** **-value**	**subterranean/non-subterranean**	***p*** **-value**
**Humerus**	0.0045/0.053	**0.001**	0.0041/0.057	**0.004**
**Mandible**	0.004/0.011	**0.001**	0.003/0.013	**0.001**

### Phenotypic trait covariation

Results from the PLS analyses are summarized in Table 3. We found that the humerus and the mandible are highly integrated with each other, and subterranean and non-subterranean taxa showed similarly high degree of covariation. When we repeated the PLS analyses on the size-corrected shape coordinates we did not find any difference in the significance level compared to the analyses conducted on the Procrustes coordinates (whole sample: r-pls **=** 0.90**,** *p*-value = 0.001; subterranean: r-pls **=** 0.87, *p*-value = 0.007; non-subterranean: r-pls **=** 0.92, *p*-value = 0.003).

**Table 3:** Phenotypic trait covariation values and associated *p*-values. In bold are reported the significant results

Morphological covariation		
	r-pls	*p*-value
Whole sample. Humerus / mandible	0.91	**0.001**
Subterranean. Humerus / mandible	0.88	**0.002**
Non-subterranean. Humerus / mandible	0.94	**0.005**

Table 4 summarises the result of the PLSs effect sizes comparison and of the MA analyses. Overall, subterranean and non-subterranean taxa displayed non-significant differences in both strength and direction of covariation.

**Table 4:** Comparison of strength and direction of phenotypic trait covariation

Covariation strength	Effect sizes	*p*-value
Humerus / mandible	subterranean = 3.02/non-subterranean = 2.58	0.205
Mandible	subterranean = 3.91/non-subterranean = 2.73	0.266
Covariation trajectories	Slopes	*p*-value
Humerus / mandible	subterranean = 0.38/non-subterranean = 0.55	0.19
Mandible	subterranean = 0.78/non-subterranean = 1.03	0.07

**Fig. 5:**
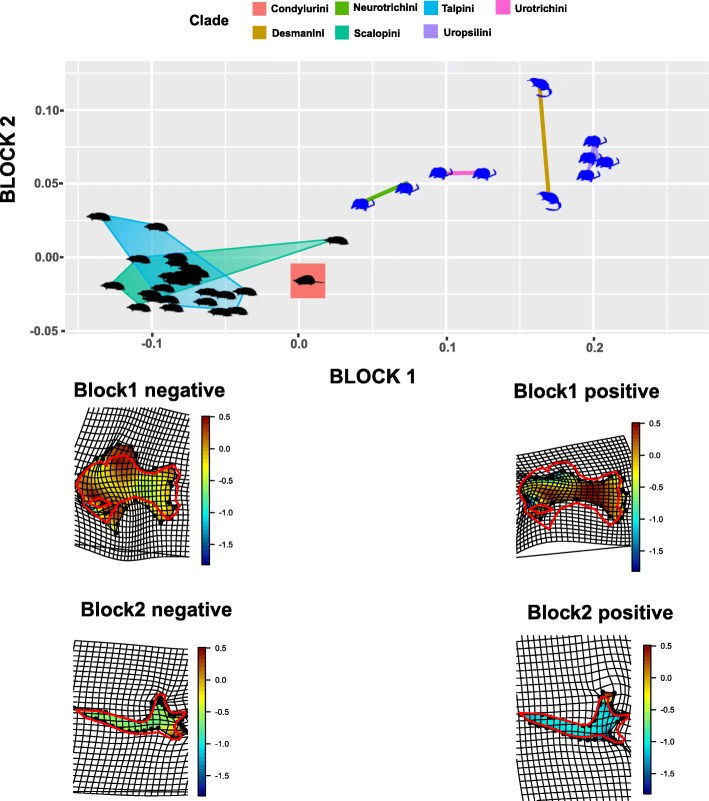
Plot of partial least squares (PLS) scores from block1 versus block2 along the first set of PLS axes for the humerus and mandible. Deformation grids refer to positive and negative extremes of the axes. Deformation grids shows color according to log(det(jacobian)) coming from the two-dimensional Thin Plate Spline first derivative evaluated within the body. Values < 0 indicate that, with respect to the source (here the sample’ consensus), the target (here the PC’s extremes) experiences a reduction in the local area, while values > 0 indicate an enlargement. Black silhouettes represent fossorial species, blue silhouettes represent non-fossorial species. Animal silhouettes were available under Public Domain license at phylopic (http://phylopic.org/). Specifically, shrew-mole (http://phylopic.org/image/822c549b-b29b-47eb-9fe3-dc5bbb0abccb/), this image is available for reuse under the Public Domain Dedication 1.0 license; *Talpa europaea* (http://phylopic.org/image/0465d81c-0def-4478-af15-a075d472e957/), this image is available for reuse under the Public Domain Dedication 1.0 license; *Condylura* (http://phylopic.org/image/8b656a93-ecf3-4985-8f4d-1f5c032d1a27/), available for reuse and under the Creative Commons Attribution 3.0 Unported (https://creativecommons.org/licenses/by/3.0/) image by Didier Escouens (vectorized by T. Michael Keesey). Desmans (http://phylopic.org/image/f6146c1d-874f-45a8-9d5c-eff0d9df0802/), this image is available for reuse under the Public Domain Dedication 1.0

The results of PLS analyses are reported in Fig. 5. In the PLS plot of mandible and humerus the subterranean species placed at negative extremes of the two PLS axes, while non-subterranean taxa placed at positive extremes. At negative extremes the humerus showed the robust humeral configuration with the enlarged proximal region, while the mandible showed a thinner horizontal ramus, a shorter coronoid process and an enlarged angular process. At positive extremes the humerus showed the slender configuration with reduced proximal region, while the mandible showed a robust horizontal ramus, an elongated coronoid process and reduced angular process.

### Global integration

For both humerus and mandible the partial warp variance drops faster than the bending energy rises. The resulting slope of − 1.31 for the humerus suggests an integrated pattern. The same holds for the mandible where the regression slope of − 2.04 indicates a highly integrated pattern. We did not find any significant difference between subterranean and non-subterranean species in the degree of humeral integration (regression slopes: − 1.28; − 1.25, respectively; *p*-value = 0.573). Again, we did not find any significant difference between subterranean and non-subterranean species in the degree of mandibular integration (regression slopes: − 1.99; − 2.07, respectively; *p*-value = 0.642).

### Phylogenetic non-independence and phylogenetic signal

Phylogenetic signal in the aligned Procrustes coordinates is high for both the humerus and the mandible. Similar results were obtained when using both morphological and molecular phylogenetic hypotheses (see Table 5). The correlation coefficient was non-significant for the phylogenetically-informed version of PLS when using both morphological and molecular phylogenetic hypotheses (r = 0.563, *p*-value = 0.268; r = 0.565, *p*-value = 0.216; respectively). However, we found the humerus and mandible to be significantly correlated in subterranean moles when using both morphological and molecular phylogenetic hypotheses (r = 0.895, *p*-value = 0.016; r = 0.893, *p*-value = 0.017; respectively), whereas correlation was not significant in non-subterranean moles (r = 0.726, *p*-value = 0.123; r = 0.718, *p*-value = 0.124; respectively). Similar results were obtained when we removed the size effect from the shape data when using both morphological and molecular phylogenetic hypotheses. The correlation was significant for subterranean moles (r = 0.815, *p*-value = 0.018; r = 0.895, *p*-value = 0.016; respectively), whereas correlation was not significant in non-subterranean moles (r = 0.706, *p*-value = 0.146; r = 0.701, *p*-value = 0.138; respectively).

**Table 5:** K_mult_ statistics and relative *p*-value for humerus and mandible. In bold are reported the significant results

	Cladistic phylogeny	Molecular phylogeny	*p*-vlaue
Humerus	K_mult_ = 1.421	K_mult_ = 1.214	**0.001**
Mandible	K_mult_ = 0.814	K_mult_ = 0.726	**0.001**

Polly et al., 2013 noted that when running the phylogenetic version of the PLS analysis, shape changes associated with PLS axes could not be biologically interpretable, possibly reflecting the removal of the phylogenetic component associated with the morphological adaptive signal (see Additional file 2 for further details). Therefore, we will present and discuss only the PLS distribution, and associated shape changes, obtained prior to phylogenetic correction.

## Discussion

During their evolution, talpid moles diversified into a number of ecological niches and geographical areas [12, 18, 19, 71, 72]. The colonization of the subterranean environment is certainly the largest ecological transition ever experienced by the clade. It represents one of the most remarkable suites of adaptation showed by any mammalian group [17, 73]. Our study demonstrates that the transition to subterranean environments resulted in dramatically reduced shape disparity in both the humerus and the mandible of subterranean species (see Figs. 3 and 4). The subterranean ecotope is structurally simple, relatively stable and highly demanding in terms of locomotion [12, 13]. These features require a high degree of specialization, forcing species within a narrow ecological niche [12]. Therefore, unrelated species evolving in this simple, but highly demanding environment are expected to display a high degree of phenotypic convergence [13].

Our results suggest that the two fully subterranean mole tribes (Talpini and Scalopini) have humeral [20, 39] and mandibular shape variation significantly reduced by functional constraints that are imposed by their highly specialized lifestyle.

The humerus of subterranean species is highly adapted for digging. Its evolution is characterized by slow evolutionary rates, convergent allometric trajectories among different talpid clades and, overall, a strong conservatism, as suggested by the presence of a strong phylogenetic signal [19–21, 39]. The same considerations hold true for the mandible, where the subterranean species display remarkable morphological similarity (see Fig. 4) despite the different degree of dental reduction in the two tribes [74]. The diet of subterranean moles mainly includes earthworms (Annelida, up to 90% in some species; [75–78]), while non-subterranean moles have a broader range of food items including beetles, fish, crustaceans, plant material and seeds [45, 79–81]. However, the mandible showed a lower, yet significant, phylogenetic signal. This could be a consequence of the evolution of different adaptive strategies within a monophyletic group. Examples are represented by the evolution of hypsodont dentition in the urotrichine genus *Urotrichus* (which contributes to the ecological separation form *Dymecodon* [45]), and the independent evolution of the semi-aquatic lifestyle in desmans and *Condylura*, which separate them from the other talpids along PC2. Furthermore, the star-nosed mole (*Condylura*) displays a highly derived oral apparatus designed for underwater high-velocity food consumption [80]. The derived condition in *Condylura* is somewhat typical for mammals adapted to a semi-aquatic lifestyle [3, 82–84].

We did not find any significant difference between subterranean and non-subterranean taxa in either strength or direction of trait covariation. We found a strong correlation between the humerus and the mandible in the whole sample and in the per-group separated analyses. Though, when accounting for phylogeny on the whole sample, we found that the correlation between the humerus and the mandible to be no longer significant. This could highlight the presence of a strong phylogenetic structure suggesting that the humerus and the mandible could have followed separated evolutionary pathways. However, we found the humerus and mandible to be significantly correlated only in subterranean moles, when accounting for shared ancestry. This result suggests, on the one hand, how the subterranean species’ shift into a different ecological niche was accompanied by concerted changes in both locomotion and dietary patterns. On the other hand, it reflects how the lack of covariation between the humerus and mandible of non-subterranean species might imply an independent evolution of the two structures, likely subject to lower environmental constraints or different functional trade-offs. In this case, greater evolutionary lability could have played a role in the adaptation of non-subterranean species to different environments [1, 4, 5, 85]. However, similar patterns of trait covariation between subterranean and non-subterranean taxa suggest the presence of a strong phylogenetic conservatism in talpids. Strong trait covariation is usually associated with reduced phenotypic variability, while modularity, by breaking down patterns of covariation between structures, might increase the number of possible axes of variation along which the phenotypes might diversify [5]. However, module covariation can generate either more or less diversity according to the selective pressures acting on the principal axes of variation [1, 5, 86]. In the context of the present study, it is possible that high trait covariation might have constrained talpid evolution along lines of least evolutionary resistance, meaning that developmental processes might offer simple pathways to generate variation [87, 88]. It has been demonstrated that subterranean moles display an early autopodial chondrification as compared to non-subterranean taxa [89, 90]. This evidence suggests that subterranean moles might experience high levels of phenotypic covariation earlier during their ontogenetic development, resulting in the reduced morphological variability showed at the adult stage. A similar pattern was observed in marsupials where high integration could enhance the effect of the intense functional demand for continuous suckling earlier in their development, hence resulting in limited variability of the marsupial oral apparatus [1]. Finally, we did not find any significant impact of size on the patterns of trait covariation in talpids, as well as on their morphological disparity. These results suggest that allometry could have played a major role in shaping talpids rates of morphological evolution [39], without significantly impacting the patterns of trait covariation. Nonetheless, changes in shape and proportions may represent another potential factor in restricting disparity [29, 30, 41, 91]. Sansalone et al. [39] demonstrated that the convergence of allometric trajectories between subterranean moles (Talpini and Scalopini) constrained the humeral shape to a restricted region of the morphospace. In this case, the response to a strong selective pressure may have resulted in the evolution of high covariation and evolutionary allometry. A consequence of these mechanisms is that subterranean mole morphology is extremely constrained [19, 20, 39].

## Conclusions

Our study showed that the transition to the subterranean ecotope resulted in a significant loss of disparity in the subterranean clades compared to the non-subterranean species, probably triggered by high morphological covariation. We showed that a strong phylogenetic conservatism in the covariation patterns (strength and direction) might have played a fundamental role in constraining the axes of variation along which subterranean moles were able to attain the high degree of phenotypic specialization necessary to colonize the subterranean environment.

## Additional files

Additional file 1: Sampling details. (XLSX 54 kb)

Additional file 2:  Supplementary methods and results. (DOCX 316 kb)

Additional file 3: Phylogenetic tree calibration data. (XLSX 31 kb)

## Data Availability

The datasets supporting the conclusions of the present study are available at 10.25952/5bbbddb2d3522

## Abbreviations

ANOVA: Univariate analysis of variance.
BEN: Bending energy
bgPCA: between-group Principal Component Analysis
CS: Centroid Size
GPA: Generalized Procrustes Analysis
Institutional abbreviations: BSPG Bayerische Staatssamlung Für Paläontologie und Geologie
ISEZ-PAN: Instytut Systematyki i Ewolucji Zwierząt-Polskiej Akademii Nauk
J: Jacobian
LACM: Los Angeles County Museum
MA: Major Axis
PLS: Partial Least Squares
PRD: Procrustes Distances
UCMP: University of California Museum of Paleontology
