# Supplementary material for: Impact of transition to a subterranean lifestyle on morphological disparity and integration in talpid moles (Mammalia, Talpidae)
Source: BMC Evol Biol. 2019 Sep 12;19:179. doi: 10.1186/s12862-019-1506-0 (PMC6739959; doi:10.1186/s12862-019-1506-0)
Supplement: Supplementary file 2 — Supplementary methods and results. (DOCX 316 kb) [file 12862_2019_1506_MOESM2_ESM.docx]

**Additional file 2 to:**

**Impact of subterranean transition on morphological disparity and integration in talpid moles (Mammalia, Talpidae)**

Gabriele Sansalone, Paolo Colangelo, Anna loy, Pasquale Raia, Stephen Wroe, Paolo Piras

**Morphological integration accounting for phylogeny**

Phylogenetic comparative methods are often employed for studying the relationship between phenotypes and environment, however not all environmental effects on morphology arise from non-phylogenetic sources [69]. When the phylogenetic changes reflect an adaptive change (sensu [70]), the functional component of variation is strongly associated with the phylogenetic history. In other words, functional changes are perfectly correlated with phylogeny [69]. Therefore, removing phylogenetic correlations from data could precisely remove that component of shape variation that is relevant to adaptation. In such a cases, accounting for phylogenetic effects could obscure the evolutionary relationships among phenotypic traits [69,71]. Similarly, the removal of phylogenetic covariance in analysis of modularity and/or integration should be carefully considered, in particular when the genetic and developmental component of morphological integration is particularly strong and, hence, more likely to be highly correlated to the tree topology [69]. In fact, when we examined the resulting plot of the first PLS axes we further noticed that removing the phylogenetic structure returned a biologically uninformative context (see Additional figure 3). In example, at negative values of both axes highly fossorial species (Talpini) were associated with a slender humeral configuration, while at positive values non-fossorial species were associated with the robust humeral configuration typical of the highly fossorial taxa.

**Additional figure 3.** Plot of phylogenetic partial least squares (PLS) scores from block1 versus block2 along the first set of PLS axes for the humerus and mandible. Deformation grids refer to positive and negative extremes of the axes.

******

**Deflated Procrustes analysis**

The deflation procedure, introduced by Bookstein (2015), relies on the concept of self-similarity that, by analogy with studies of temporal series in modern palaeobiology, can be identified with the finding of random walks. These self-similar spatial processes can be generated by an algebraic manipulation of partial warps within the standard toolkit of thin plate spline (Bookstein, 2015). The deflation procedure can be summarized by the following algorithm:

$defl$ *= Pmean +* $\sum_{l=1}^{k-3} \sqrt{\frac{E1}{El}}\left( Wl \cdot Pdist \right)Wl$

Here *Pmean* is the mean of the Procrustes distribution *Pdist*, the quantity *(Wl* $\cdot$ *Pdist)Wl* are the partial warp scores scaled by the inverse of the square root of their specific bending energy (with respect of the largest partial warp *l*=1).

The deflation of partial warps allows the emergence of integrated (large scale) or disintegrated (small scale) patterns against the null hypothesis of self-similarity. These patterns can be parameterised by performing the log-log regression of non-affine partial warps variance on the bending energy.

However, to our knowledge, a series of published studies which used the log-log regression of partial warps variance on the bending energy did not perform the “deflation” of the partial warps (Young et al., 2017; Evans et al., 2017; Piras et al., 2017; Profico et al., 2017; Arlegi et al., 2018). The deflation algorithm is a necessary assumption without which results can’t be properly interpreted. Hence, we strongly recommend its application and, at this purpose, we implemented a function in the R statistical environment, which is available upon request from the corresponding author (G. Sansalone).

**References**

Bookstein, F. L. "Integration, disintegration, and self-similarity: characterizing the scales of shape variation in landmark data." *Evolutionary biology* 42.4 (2015): 395-426.

Young, N. M., et al. "Craniofacial diversification in the domestic pigeon and the evolution of the avian skull." *Nature ecology & evolution* 1.4 (2017): 0095.

Evans, K. M., et al. "Why the short face? Developmental disintegration of the neurocranium drives convergent evolution in neotropical electric fishes." *Ecology and evolution* 7.6 (2017): 1783-1801.

Profico, A., et al. The evolution of cranial base and face in Cercopithecoidea and Hominoidea: Modularity and morphological integration. *American journal of primatology*79.12 (2017): e22721.

Piras, P., et al. Homeostatic Left Heart integration and disintegration links atrio-ventricular covariation’s dyshomeostasis in Hypertrophic Cardiomyopathy. *Scientific reports* 7.1 (2017): 6257.

Arlegi, M., A. Gómez‐Robles, and A. Gómez‐Olivencia. Morphological integration in the gorilla, chimpanzee, and human neck. *American journal of physical anthropology* 166.2 (2018): 408-416.

**Reconstruction of the phylogenetic relationships within the family Talpidae**

The following characters list is derived from Hooker (2016). Characters following or modified from Sánchez-Villagra *et al*. (2006) have also been used by Schwermann and Thompson (2015) with additions of their own, also cited here. N.B. The ‘0’ state is primitive unless otherwise indicated. Multistate characters are ordered unless otherwise stated. The cladistics analysis has been performed in order to reconstruct the relationships within the family Talpidae. The matrices have been analysed using PAUP 4.0 a147 (Swofford 2002) using a heuristic search and stepwise addition, with a random addition sequence of 1000 replicates. Bremer supports (Decay indices; Bremer 1994) were calculated using TNT 1.1 (Goloboff *et al*. 2008). For the sake of visualization, we showed the strict consensus tree computed on the maximum parsimony trees found (see Figure 1A). The maximum parsimony analysis performed using the character matrix resulted in three maximum parsimony trees with a consistency index (CI) of 0.3571 (excluding uninformative characters) and a retention index (RI) of 0.6308.

## Teeth

1. First lower incisor, I_1_, present (0); absent (1). (Sánchez-Villagra *et al*. 2006, character 1, coding reversed. N.B., the first lower incisor of soricids is identified as I_1_ following Lopatin, 2006).

2. Third upper incisor, I^3^, present (0); absent (1). (Sánchez-Villagra *et al*. 2006, character 2, coding reversed.

3. Third lower incisor, I_3_, present (0); absent (1). (Sánchez-Villagra *et al*. 2006, character 3, coding reversed. *Uropsilus*, *Urotrichus* and *Dymecodon* coded for presence following Ziegler, 1971).

4. DP^1^ present (0); absent (1). (Sánchez-Villagra *et al*. 2006, character 4, coding reversed).

5. DP_1_ present (0); absent (1). (Sánchez-Villagra *et al*. 2006, character 5, coding reversed).

6. P^2^ present (0); absent (1). (Sánchez-Villagra *et al*. 2006, character 6, coding reversed).

7. P_2_ present (0); absent (1). (Sánchez-Villagra *et al*. 2006, character 7, coding reversed).

8. P^3^ with two or more roots (0); single-rooted (1); absent (2). (Sánchez-Villagra *et al*. 2006, characters 8 and 9).

9. Relative size (height and width) of I^1^ and upper canine: canine larger (0); I^1^ larger (1). (Sánchez-Villagra *et al*. 2006, character 10, coding reversed).

10. P^4^, number of roots: three (0); two (1); one (2). (Sánchez-Villagra *et al*. 2006, character 11, coding reversed).

11. Upper canine, number of roots: one (0); two (1). (Modified from Sánchez-Villagra *et al*. 2006, character 13. N.B. *Asioryctes* has 2, *Ukhaatherium* has one: Kielan-Jaworowska 1981; Novacek et al. 1997. One treated as primitive).

12. Upper canine: caniniform, without posterior crest (0); caniniform with posterior crest (1); premolariform (2). (Sánchez-Villagra *et al*. 2006, character 15). UNORDERED.

13. M^2^ relative height of buccal cusps: subequal or paracone >metacone (0); metacone>paracone (1). (Sánchez-Villagra *et al*. 2006, character 14, coding reversed).

14. M^1^ metacone not expanded distolingually (0); expanded (1). (Sánchez-Villagra *et al*. 2006, character 16).

15. M^2^ paraconule present (0); absent (1). (Sánchez-Villagra *et al*. 2006, character 17, coding reversed).

16. M^2^ hypocone: absent (0); present distolingual with talon shelf (1); present mesially positioned with talon shelf (2) (Fig. 1C); mesially positioned without talon shelf (or if not distinguished, position on postprotocingulum) (3) (Fig. 1E). (Sánchez-Villagra *et al*. 2006, character 18, but homology corrected from metaconule to hypocone, following Butler, 1988, and states 2 and 3 added).

17. M^1^ mesostyle: absent (0); present single (1); present double, close to eachother (2); present double, separated by deep valley (3). (Sánchez-Villagra *et al*. 2006, character 19).

18. M^2^ mesostyle: absent (0); present single (1); present double, close to eachother (2); present double, separated by deep valley (3). (Sánchez-Villagra *et al*. 2006, character 20).

19. M^2^ postmetacrista and preparacrista subequal (0); postmetacrista longer (1). (Sánchez-Villagra *et al*. 2006, character 21, coding reversed. Both Sánchez-Villagra *et al*. 2006 and Schwermann and Thompson 2015 have used metacrista and paracrista respectively for these crests).

20. Anterior accessory cuspid of M_2-3_ (Hutchison 1968, fig. 6): absent (0); present (1). (Modified from Sánchez-Villagra *et al*. 2006, character 22.

21. Lower molar precingulid: present (0); absent (1). (Sánchez-Villagra *et al*. 2006, character 23, coding reversed. N.B., soricids coded for presence contra Sánchez-Villagra *et al*. 2006: pers. obs.).

22. Relative heights of M_1_ entoconid and metaconid: metaconid > entoconid (0); subequal (1). (Sánchez-Villagra *et al*. 2006, character 24).

23. M_1-2_ talonid notch: present (0); absent (1). (Sánchez-Villagra *et al*. 2006, character 25, coding reversed).

24. Position of attachment of M_2_ cristid obliqua to back of trigonid: lingual (0); central (1) (PRIMITIVE); buccal (2). (Modified from Sánchez-Villagra *et al*. 2006, character 26).

25. M_1-2_ talonid: with central hypoconulid on postcristid (0); nyctalodont (1); myotodont (2).

26. Upper premolar row: without gaps (0); with gaps (1). (Sánchez-Villagra *et al*. 2006, character 28. N.B. soricids coded for without).

27. Lower premolar row: without gaps (0); with gaps (1). (Sánchez-Villagra *et al*. 2006, character 42. N.B. soricids coded for without).

28. Contact between I^2^ and I^1^: present (0); absent (1). (Sánchez-Villagra *et al*. 2006, character 29, coding reversed).

29. Upper canine: taller than I^1^ (0); subequal to I^1^ (1); shorter than I^1^ (2). (Sánchez-Villagra *et al*. 2006, character 30, coding reversed).

30. Upper canine length: ≤ width (0); > width (1). (Sánchez-Villagra *et al*. 2006, character 31).

31. Height of P^4^: < upper canine (0); = upper canine (1); > upper canine (2). (Motokawa 2004, character 26, coding reversed).

32. P^4^ protocone large, mesiolingual of paracone (0); small to insignificant, lingual to distolingual of paracone (1). (Replaces Sánchez-Villagra *et al*. 2006, character 33, to which it adds data).

33. P^4^ parastyle: obvious (0); inconspicuous (1). (Sánchez-Villagra *et al*. 2006, character 34).

34. M^2^ length: c.= M^1^ length (0); < M^1^ length (1). (Sánchez-Villagra *et al*. 2006, character 35).

35. Length of M^3^: more than half M^1^ (0); less than half (1). (Sánchez-Villagra *et al*. 2006, character 36).

36. Crown area of M^3^: = or > P^4^ (0); < P^4^ (1). (Sánchez-Villagra *et al*. 2006, character 37).

37. DP^4^/_4_: functional (0); non-functional (1). (Sánchez-Villagra *et al*. 2006, character 38).

38. Posterior cingulum cusp of I_2_: absent (0); present (1). (Sánchez-Villagra *et al*. 2006, character 39, coding reversed).

39. P_4_ paraconid: present (0); absent (1). (Sánchez-Villagra *et al*. 2006, character 40).

40. P_4_ talonid: absent (0); unbasined with hypoconulid (1) (PRIMITIVE); basined with hypoconid and hypoconulid (2). (Modified from Sánchez-Villagra *et al*. 2006, character 41).

41. P_4_ metaconid: present (0); absent (1). N.B. Asioryctitheres lack a P_4_ metaconid, but its presence is widespread in other primitive eutherians, so presence is treated as primitive here.

42. M^1^ preparacrista: present (0); absent (1).

43. Length of M_1_ c.= P_4_ (0); longer but less than twice as long as P_4_ (1); more than twice as long as P_4_ (2). (Sánchez-Villagra *et al*. 2006, character 43).

44. Length of M_2_: < M_1_ (0); M_1_ and M_2_ subequal (1) (PRIMITIVE); > M_1_ (2). (Sánchez-Villagra *et al*. 2006, character 44).

45. Length of M_3_: > or subequal to M_1_ (0); < M_1_ (1). (Sánchez-Villagra *et al*. 2006, character 45, coding changed).

46. M_2_ metastylid: absent (0); present (1). (Sánchez-Villagra *et al*. 2006, character 46).

47. M_2_ talonid width: < trigonid (0); subequal to trigonid (1). (Sánchez-Villagra *et al*. 2006, character 47, coding reversed).

48. M_1_ crown height, unworn to lightly worn: height of protoconid as percentage of tooth length: >90% (0); <90% (1). (Sánchez-Villagra *et al*. 2006, character 70, related crown height to dentary height. Here, both measurements are from the tooth, reducing the influence of other variables).

## Cranium

49. Anterior nasal tip in lateral view: reaches level of incisors (0); level of canines (1); posterior to the posterior margin of canines (2). (Sánchez-Villagra *et al*. 2006, character 48).

50. Anterior extremity of incisive foramen: reaches level of I^3^ or more posterior (0); reaches level of I^2^ (1); anterior to the anterior margin of the I^2^ (2). (Sánchez-Villagra *et al*. 2006, character 49, polarity reversed). N.B. Not known in Asioryctitheria or other primitive eutherians; polarity taken from *Leptictis* (Novacek 1986).

51. Incisive foramina: small, anteroposterior length shorter than length of M^2^ (0); large, ≥ length of M^2-3^ (1). (Sánchez-Villagra *et al*. 2006, character 63). N.B. Not known in Asioryctitheria or other primitive eutherians; polarity taken from *Leptictis* (Novacek 1986).

52. Anterior extremity of anterior/major palatine foramina reaches: level of M^2^ (0); level of M^1^ (1); level of P^4^ (2). (Sánchez-Villagra *et al*. 2006, character 50, coding reversed).

53. Position of posterior border of infraorbital foramen relative to upper molar row: anterior to or at border of M^1^ and M^2^ (0); above M^2^ or more posterior (1). (Modified from Sánchez-Villagra *et al*. 2006, character 68).

54. Posterior margin of anterior root of zygomatic arch in ventral view extends: to M^3^ (0); to M^2^ (1). (Sánchez-Villagra *et al*. 2006, character 51; polarity reversed).

55. Zygomatic plate: dorsoventrally deep (0); shallow (1). (Motokawa 2004, character 6, fig. 2, coding reversed).

56. In dorsal view, location of contact of zygomatic arch with braincase: medial to or at midpoint of anterior margin of braincase (0); at lateral portion of anterior margin of braincase (1); absent (2). (Modified from Sánchez-Villagra *et al*. 2006, character 55, coding reversed). Polarity based on ingroup commonality. UNORDERED.

57. Posterior margin of the infraorbital foramen: extends to M^1^ level or more anterior (0); extends to M^2^ level (1); extends to M^3^ level or more posterior (2). (Reworded and modified from Sánchez-Villagra *et al*. 2006, character 56; Motokawa 2004, character 12).

58. Zygomatic arch complete (0); broken (1). (Sánchez-Villagra *et al*. 2006, character 52).

59. Position of lacrymal foramen: posterior to infraorbital foramen (0); just dorsal or dorsal at the level of the middle portion of infraorbital canal (1); dorsal, just anterior to anterior border of infraorbital canal (2). (Sánchez-Villagra *et al*. 2006, character 62).

60. Foramen “I” in maxilla or premaxillary-maxillary suture: absent (0); present (1). (Sánchez-Villagra *et al*. 2006, character 64). Polarity based on ingroup commonality.

61. Anterior projection of mastoid: well-developed, projecting laterally (0); weak to absent (1). (Sánchez-Villagra *et al*. 2006, character 58).

62. Anterior process of “mastoid process”: below root of zygomatic arch (0); in line with root (1); above root of zygomatic arch (2). (Sánchez-Villagra *et al*. 2006, character 67, coding reversed).

63. Position of posterior extremity of auditory bulla in ventral view: anterior to the anterior process of the “mastoid process” (0); in a similar position (1) PRIMITIVE; posterior (2). (Sánchez-Villagra *et al*. 2006, character 57).

64. Maximal/minimal diameter of fenestra ovalis: <2.5 (0); >2.5 (1). (Sánchez-Villagra *et al*. 2006, character 71).

65. Stapes footplate: not bullate (0); bullate (1). (Sánchez-Villagra *et al*. 2006, character 72). Polarity based on ingroup commonality.

66. Bony canal surrounding stapedial artery traversing the stapedial foramen: absent (0); canal partially or totally ossified (1). (Sánchez-Villagra *et al*. 2006, character 73). Polarity based on ingroup commonality.

67. Number of mental foramina: three or more (0); two (1); one (2). (Sánchez-Villagra *et al*. 2006, character 65, coding reversed).

68. Posterior mental foramen between P_3_ and P_4_ (0); between P_4_ and M_1_ (1); below M_1_ (2).

69. Posterior tip of the angular process of the dentary: anterior to condyle (0); approximately level with the condyle (1); posterior to the condyle (2). (Sánchez-Villagra *et al*. 2006, character 60).

70. Dentary angular process: plate-like (0); rod-like (1). (Modified from Sánchez-Villagra *et al*. 2006, character 66; coding changed for *Condylura*, where it is narrow but still plate-like, not rod-like).

71. Position of mandibular condyle: between angular process and coronoid tip, but nearer angular process (0); at midpoint between upper sigmoid notch and coronoid tip or nearer coronoid tip (1). (Modified from Sánchez-Villagra *et al*. 2006, characters 59 and 61).

## Postcranial, axial

72. Axis and C3: not ankylosed (0); ankylosed (1). (Sánchez-Villagra *et al*. 2006, character 85).

73. Axis neural spine: cranio-caudally orientated keel (0); simple knob (1). (Sánchez-Villagra *et al*. 2006, character 86, coding reversed).

74. C6 transverse process posterior extension: does not reach C7-T1 border (0); reaches or surpasses this border (1). (Sánchez-Villagra *et al*. 2006, character 87).

75. Number of caudal vertebrae: 20 or more (0); <20, >14 (1); 14 or fewer (2). (Sánchez-Villagra *et al*. 2006, character 88, coding reversed). Polarity based on *Eomaia* (Ji et al. 2002).

76. Sternum ventral surface: no distinct ridge (0); distinct ridge, but no keel (1); prominent keel (2). (Sánchez-Villagra *et al*. 2006, character 83). Polarity based on ingroup commonality.

77. Sternum, proportions of manubrium: length/width <1.5 (0); 1.5-3 (1); 3-4.5 (2); >4.5 (3). (Sánchez-Villagra *et al*. 2006, character 84). Polarity based on ingroup commonality.

78. Dorsal surface of manubrium: smooth (0); contains a well-defined trough (1); features a ridge, sometimes perforated by a foramen (2). (Schwermann and Thompson 2015, character 158). UNORDERED

## Postcranial, appendicular, forelimb

79. Clavicle: elongate, in some cases with strong processes directed medio-ventrally (0); semirectangular, stout (1); quadratic (length c.= width) (2). (Sánchez-Villagra *et al*. 2006, character 74). Polarity based on commonality in modern placentals.

80. Clavicle, “foramen for vein”: absent (0); present (1). (Sánchez-Villagra *et al*. 2006, character 75). Polarity based on commonality in modern placentals.

81. Clavicle, articulations: with scapula (0); with scapula and humerus (1); with just humerus (2). (Sánchez-Villagra *et al*. 2006, character 76). Polarity based on commonality in modern placentals.

82. Tetrahedral heterotopic bone wedged between ventromedial spine of the clavicle and anterior basilateral portion of the manubrium: absent (0); present (1). (Sánchez-Villagra *et al*. 2006, character 77). Polarity based on ingroup commonality.

83. Scapula, suprascapular canal through base of acromion: absent (0); present (1). (Sánchez-Villagra *et al*. 2006, character 78). Polarity based on ingroup commonality.

84. Scapula, infraspinous fossa: present (0); absent (1). (Sánchez-Villagra *et al*. 2006, character 79, coding reversed).

85. Scapula, marked teres fossa: absent (0); present (1). (Sánchez-Villagra *et al*. 2006, character 80).

86. Scapula metacromion: absent (0); present < one third the length of the spine (1); present ≥ one third the length of the spine (2). (Sánchez-Villagra *et al*. 2006, character 81).

87. Scapula, coracoid process: conspicuous (0); inconspicuous (1). (Sánchez-Villagra *et al*. 2006, character 82, coding reversed). Polarity based on *Eomaia* (Ji et al. 2002).

88. Deltoid process of humerus: absent (0); present as flange distal to the greater tuberosity (1); present as elongate hook on lateral edge of greater tuberosity (2). (Sánchez-Villagra *et al*. 2006, character 89, states corrected by Schwermann and Thompson 2015).

89. Position of humeral head: on posterior to posteromedial side of proximal end (0); lateral edge to centre of head in line with lateral edge of shaft (1); medial edge of head in line with lateral edge of shaft (2); entire head lateral to lateral edge of shaft (3). (Sánchez-Villagra *et al*. 2006, character 90, where state 3 was not scored. Some states have been recoded).

90. Orientation of humeral head: long axis of head parallel or subparallel to shaft long axis (0); long axis of head at oblique angle to shaft long axis (1). (Sánchez-Villagra *et al*. 2006, character 91).

91. Minimum width of humerus: approximately 1/9-1/10^th^ of maximum length of humerus (0); approximately 1/7^th^ (1); approximately 1/4-1/5^th^ (2); approximately 1/3^rd^ or less (3). (Sánchez-Villagra *et al*. 2006, character 92).

92. Distal end of pectoral crest of humerus: does not form pronounced and distinct process (0); forms pronounced and distinct process orientated proximo-medially (1); small but prominent process protruding at right angles to shaft (2). (Sánchez-Villagra *et al*. 2006, character 93, modified Schwermann and Thompson 2015). UNORDERED

93. Proximity of pectoral crest to lesser tuberosity: clear gap with low proximal end of pectoral process (0); narrow gap or fused to form a bicipital tunnel (1). (Sánchez-Villagra *et al*. 2006, character 94).

94. Floor of bicipital groove: straight and parallel to long axis of humerus (0); displaced medially by pectoral crest near proximal end of humerus (1). (Sánchez-Villagra *et al*. 2006, character 95).

95. Open portion of proximal half of bicipital groove: visible in anterior view (0); visible in posterior view (1). (Modified from Sánchez-Villagra *et al*. 2006, character 96, where a state 2, “not visible”, was not scored).

96. Pit for m. flexor digitorum profundus: absent (0); present (1). (Sánchez-Villagra *et al*. 2006, character 97).

97. Medial edge of humeral trochlea: sharp, ventrally projecting ridge (0); straight or low ridge (1). (Sánchez-Villagra *et al*. 2006, character 98).

98. Lateral epicondyle: present as rounded protuberance (0); forms laterally extended flange (1); has proximally directed hook (2); has spine-like proximally pointed hook (3). (Sánchez-Villagra *et al*. 2006, character 99).

99. Brachial fossa: small pit (0); cavernous excavation underlying greater tuberosity (1). (Sánchez-Villagra *et al*. 2006, character 100).

100. Crest between greater tuberosity and distal end of pectoral ridge: present (0); absent (1). (Sánchez-Villagra *et al*. 2006, character 101).

101. Trough between head of humerus and greater tuberosity: very shallow to absent (0); deep groove (1). (Sánchez-Villagra *et al*. 2006, character 102).

102. Lesser tuberosity in posterior view: lower than proximal edge of head (0); level with proximal edge of head (1) higher than proximal edge of head (2). (Sánchez-Villagra *et al*. 2006, character 103, modified Schwermann and Thompson 2015).

103. Humeral head round (0); elliptical (1). (Sánchez-Villagra *et al*. 2006, character 104).

104. Scalopine ridge, running between the medial root of the humeral head and the distal margin of the lesser tuberosity: absent or weak (0); present as a distinct ridge or shelf (1). (Schwermann and Thompson 2015, character 171).

105. Medial epicondyle, proximally elongate flange or process: absent (0); present (1). (Sánchez-Villagra *et al*. 2006, character 105).

106. Greatest length of greater tuberosity and deltoid process: relatively short, c. <1/4 length of humerus (0); longer (1). (Sánchez-Villagra *et al*. 2006, character 106).

107. Pectoral crest: single straight process parallel to humerus long axis (0); forms single curved process (1); long axis of humerus and pectoral crest have perpendicular orientation (c.90 degrees) (2). (Sánchez-Villagra *et al*. 2006, character 107).

108. Clavicular facet: absent (0); present (1). (Modified from Sánchez-Villagra *et al*. 2006 and Schwermann and Thompson, 2015, character 108, as few taxa have been coded on shape).

109. Lateral side of capitulum: not noticeably elongate (0); laterally elongate, so that capitulum has fusiform shape (1). (Sánchez-Villagra *et al*. 2006, character 109).

110. Teres tubercle of humerus: absent (0); a weak muscle scar (1); a distinct proximodistally short process (2); a distinct proximodistally elongate process (3). (combined from Schwermann and Thompson, 2015, characters 172, 173).

111. Length of olecranon process of ulna: c.= length of semilunar notch (0); longer (1). (Modified from Schwermann and Thompson, 2015, character 167 to allow incomplete *Eotalpa* ulna to be coded).

112. Ulna, radial facet: flat to concave (0); convex, steep on lateral face (1). (Includes radial capitular process: Schwermann and Thompson, 2015, character 170).

113. Ulna radial facet: confluent distally with humeral facet (0); separated distally by notch (1).

114. Ulna, proximal olecranon crest: absent (0); incipient, lateral process at level of anconal process (1) (Figs 4B, D, 5G); well-developed, but strongly oblique (2) (Fig. 5C, H); well-developed and nearly transverse (3) (Fig. 5D, E, I-J). (Modified from Schwermann and Thompson, 2015, character 166).

115. Anterior tubercle of olecranon: absent (0) (Fig. 5A); present, strong, halfway along olecranon (1) (Fig. 5B); present, strong, at proximal end of olecranon (2) (Fig. 5C); present, weak towards proximomedial end of medial olecranon crest, where it meets the edge of the triceps area of insertion (3) (Fig. 5D); subsumed where the medial crest meets the triceps area of insertion at the medial extremity of the proximal olecranon crest (4) (Fig. 5E).

116. Ulna, anconal process: weak (0); strong (1).

117. Ulna coronoid process: weak (0); strong (1).

118. Abductor fossa and posterior crest of ulna: fossa narrow and very shallow, and crest a weak ridge (0); fossa deep, forming thin plate of bone and laterally curved forming a pronounced posterior crest (1). (Schwermann and Thompson 2015, character 168, where *Uropsilus* was coded ‘1’, despite having a very narrow fossa as noted by Hutchison, 1968: 15, fig. 4. Nevertheless, the depth in *Uropsilus* is greater than in soricids and appears to begin a trend towards ever broader and deeper fossae: Hutchison, 1968, fig. 13).

119. Terminal process of the distal ulna (sensu Hutchinson 1968), defined as an elongate posteriorly projecting process: absent (0); present (1). (Schwermann and Thompson 2015, character 169).

120. Prepollex: absent (0); present as a knob (1); present, elongate, extending all along the scaphoid, but not reaching metacarpal I (2); present, extending to proximal portion of metacarpal I or beyond (3). (Sánchez-Villagra *et al*. 2006, character 110). Polarity based on commonality in modern placentals.

121. Scaphoid and lunar: not co-ossified (0); co-ossified, suture visible (1); co-ossified, suture not visible (2). (Sánchez-Villagra *et al*. 2006, character 111).

122. Cuneiform, ulno-palmar extension originating from distal portion: absent (0); present (1). (Sánchez-Villagra *et al*. 2006, character 112). Polarity based on commonality in modern placentals.

123. Small sesamoid lateral to cuneiform: absent (0); present (1). (Sánchez-Villagra *et al*. 2006, character 113). Polarity based on ingroup commonality.

124. Trapezium distinct distal arms: absent (0); present (1). (Sánchez-Villagra *et al*. 2006, character 114). Polarity based on ingroup commonality.

125. Centrale: separate (0); absent/co-ossified (1). (Sánchez-Villagra *et al*. 2006, character 115, coding reversed).

126. Pisiform: simple (0); forms a plate larger in area than the cuneiform, extending palmar to the cuneiform, unciform and ulna (1). (Sánchez-Villagra *et al*. 2006, character 116). Polarity based on ingroup commonality.

127. Proximal radial process of metacarpal I: absent (0); present (1). (Sánchez-Villagra and Menke 2005).

128. Length/width ratio of metacarpal IV: X6 (0); X4 (1); X2.5 (2); approximately as broad as long (3). (Partial overlap with Schwermann and Thompson 2015, character 174, but defined to be directly relevant to *Eotalpa*). Polarity based on *Eomaia* (Ji et al. 2002).

129. Ungual phalanges at least on fore foot: laterally compressed (0); relatively dorsoventrally compressed (1).

*Postcranial, appendicular, hind limb*

130. Fusion of acetabular area to vertebrae: absent (0); present (1). (Sánchez-Villagra *et al*. 2006, character 117). Polarity based on ingroup commonality.

131. Fusion of posterior horizontal branch of ischium to vertebrae: absent (0); transverse processes expanded but not fused to ischium (1); fused (2). (Sánchez-Villagra *et al*. 2006, character 118). Polarity based on commonality in modern placentals.

132. Pubic approach: absent (0); pubes approach one another beneath acetabulum (1); pseudosymphysis formed (2). (Sánchez-Villagra *et al*. 2006, character 119, modified Schwermann and Thompson 2015). Polarity based on commonality in modern placentals.

133. Pubic symphysis in the shape of a narrow bridge: absent (0); present (1). (Sánchez-Villagra *et al*. 2006, character 120). Polarity based on ingroup commonality.

134. Femur, greater trochanter height: level with or below head (0); higher than head (1). (Sánchez-Villagra *et al*. 2006, character 121).

135. Third trochanter: absent (0); present, distal of lesser trochanter a small short flange (1); present, level with lesser trochanter, a small short flange (2); present, level with lesser trochanter, a short but broad and robust hooked flange (3). (Modified from Sánchez-Villagra *et al*. 2006, character 122 and Schwermann and Thompson 2015, character 176).

136. Tibia and fibula: separate or with syndesmosis (0); synostosed (1).

137. Tibial distal bridge: absent (0); present (1). (Sánchez-Villagra *et al*. 2006, character 123).

138. Tibial falciform process: absent (0); proximodistal blade (1); actual laterally projecting falciform process (2). (Sánchez-Villagra *et al*. 2006, character 124).

139. Fibular lateral process: absent (0); simple lateral process (1); process with proximal head (2); process with proximal and distal heads (3). (Sánchez-Villagra *et al*. 2006, character 125).

140. Fibular posterior process: absent (0); present (1). (Sánchez-Villagra *et al*. 2006, character 126).

141. Astragalus, process on lateral side of body: absent (0); present (1). (Sánchez-Villagra *et al*. 2006, character 127).

142. Astragalar head width: narrower than body (0); as wide as body or wider (1). (Sánchez-Villagra *et al*. 2006, character 128).

143. Astragalar head, lateral side height relative to medial side: equal (0); lateral side higher (1). (Sánchez-Villagra *et al*. 2006, character 129).

144. Astragalar transverse ridge or groove proximal to trochlea: absent (0); ridge or groove (1). (Modified from Sánchez-Villagra *et al*. 2006, character 130. State 1, which is an autapomorphy of *Scalopus*, is combined with state 2).

145. Astragalar proximoventral groove for the flexor digitorum fibularis tendon: shallow groove (0); deep groove or canal (1). (Sánchez-Villagra *et al*. 2006, character 131).

146. Astragalar body proportions: mediolaterally wider (0); equidimensional (1); mediolaterally narrower (2). (Sánchez-Villagra *et al*. 2006, character 132).

147. Astragalar medial trochlear ridge orientation: proximodistal (0); proximally more lateral (1). (Sánchez-Villagra *et al*. 2006, character 133).

148. Distal end of astragalar lateral trochlear ridge ends on the distal end of the body (0); body is longer (1). (Sánchez-Villagra *et al*. 2006, character 134, but coded in reverse).

149. Astragalar medial plantar tuberosity: does not protrude medially beyond medial trochlear ridge (0); protrudes medially (1). (Sánchez-Villagra *et al*. 2006, character 135).

150. Astragalar neck angle with trochlea: large angle (0); small angle (1). (Sánchez-Villagra *et al*. 2006, character 136, coding reversed).

151. Astragalar proximoventral groove does not protrude proximally in dorsal view (0); protrudes proximally (1). (Sánchez-Villagra *et al*. 2006, character 137).

152. Calcaneum, sustentacular facet dimensions: mediolaterally larger (0); equidimensional, round to square (1); proximodistally longer (2). (Sánchez-Villagra *et al*. 2006, character 138).

153. Calcaneum, peroneal process distal extent: proximal of or level with cuboid facet (0); protrudes distally (1). (Sánchez-Villagra *et al*. 2006, character 139).

154. Calcaneum, peroneal process lateral extent: protrudes laterally (0); does not protrude laterally (1). (Sánchez-Villagra *et al*. 2006, character 140, polarity reversed).

155. Peroneal process position: lateral to calcaneocuboid facet (0); dorsolateral to calcaneocuboid facet (1). (Sánchez-Villagra *et al*. 2006, character 141).

156. Calcaneal cuboid facet, major axis: mediolaterally larger (0); equilateral axes (1); dorsoventrally larger (2); dorsoventrally much larger (3). (Sánchez-Villagra *et al*. 2006, character 142).

157. Ectal facet: without concave proximal extension (0); with concave proximal extension (1). (Sánchez-Villagra *et al*. 2006, character 143).

158. Peroneal process and sustentaculum proximodistal lengths: equal or peroneal process longer (0); peroneal process shorter (1). (Sánchez-Villagra *et al*. 2006, character 144, polarity reversed).

159. Ectocuneiform medial canal: absent (0); present (1). (Sánchez-Villagra *et al*. 2006, character 145).

160. Navicular: tibial tuber weak to absent (0); short (1); long (2). (reworded from Sánchez-Villagra *et al*. 2006, character 146). N.B. primitive state is based on *Eomaia* (Ji et al. 2002), as the navicular is not known in Asioryctitheria.

161. Navicular ventral articular area: absent (0); ventral facet smaller than that on tuber (1); ventral and tuber facets subequal (2). (Sánchez-Villagra *et al*. 2006, character 147).

162. Navicular shape in dorsal view: mediolaterally wider (0); proximodistally longer (1). (Sánchez-Villagra *et al*. 2006, character 148).

163. Cuboid, medial proximal process: absent (0); present (1). (Sánchez-Villagra *et al*. 2006, character 149).

164. Cuboid ventrolateral tunnel: absent (0); present (1). (Sánchez-Villagra *et al*. 2006, character 150).

165. Cuboid proximal surface proximodistal location relative to that of the navicular: cuboid surface more distal (0); equal (1). (Sánchez-Villagra *et al*. 2006, character 151, polarity reversed).

166. Prehallux: absent or not in contact with entocuneiform and navicular (0); in contact (1). (Sánchez-Villagra *et al*. 2006, character 152).

167. Metatarsal I: with symmetrically opposite proximal and distal articulations (0); distal articulation twisted laterally (1).

168. Length of metatarsal III relative to calcaneum: much longer, at least 1.31 times longer (0); between 1.16 and 1.01 times longer (1); shorter (2). (Sánchez-Villagra *et al*. 2006, character 153).

169. Metatarsal IV distal extent relative to M/T III: about equal or M/T IV shorter (0); M/T IV extends beyond M/T III (1). (Sánchez-Villagra *et al*. 2006, character 154).

170. Metatarsal V proximal lateral process: absent (0); terminal (1); subterminal (2). UNORDERED.

171. Metatarsal V cuboid facet: present, large (0); present, small (1); absent (2).

**APPENDIX S2. CHARACTER-TAXON MATRIX USED IN THE CLADISTIC ANALYSIS**

***Uropsilus***

1100000110 0010021101 0101200120 0101100101 1111101101 0001110011 1210001201 0011010000 1000010111 000100?000 0100001??2 1000000100 2000000000 00011101?? 1000000001 0000120000 11101??001 0

***Desmana moschata***

0000000110 1010033311 0000200120 0100001010 1110101102 1101111010 0201010220 1001010000 2010121111 1011010100 1210101103 1003300100 0010001100 10113102?1 1011020011 1210130101 1011110010 1

***Galemys pyrenaicus***

0000000010 1000033301 1000200110 1100001101 1111101102 1111111011 0200?10210 0011010000 2000121110 1011010100 1210101113 1003300102 0000001100 1011310231 1011021011 1210130101 10111?0010 1

***Scaptonyx fusicaudus***

0010000002 1000032111 1010211001 2100001001 1112001001 0110102011 112??02220 0011121010 2000101210 2211110211 1210101113 ?0?1???102 011000?210 1001210121 1??0?00001 1????????? ??1??1?11? ?

***Talpa europaea***

0000000000 1100131211 1001200001 2100001001 1112100110 0110102021 1120011120 0000222120 2001101230 2011110310 0110112113 1113311113 1101010311 0100110231 1010001001 0100020111 2011110102 0

***Scaptochirus moschatus***

0000011000 1110132211 1001210001 2110001001 1111101010 0101111011 1000011200 0000?22121 2001101230 3011110310 0110112113 11?3???113 110101??11 2100110231 0110001001 02????0??? ???????21? ?

***Euroscaptor***

0000000000 1100032211 1001200001 2100001011 1111000000 0110101020 111?1?1110 0000222121 2001101220 2011110310 0110112113 1113311113 010101031? ???011?231 0110001011 1200120111 1011111112 0

***Mogera***

0010000000 1110032211 1001200001 2100001001 1111101000 0110001021 1100111110 0100222121 2001101230 2011110310 0110112113 1113411113 1101010311 2200110231 1011001001 1100120012 1011110112 0

***Parascaptor leucura***

0000010000 1100032211 1000210001 2100001001 1111100100 0110102011 1?1???2110 1100221121 2001101230 2011110310 0110112113 1113311113 1101000311 0200110231 ?1?0000?0? 01???????? ?????11202 0

***Urotrichus talpoides***

1000111010 0000031101 1111200120 0100000111 1111101102 0111001010 1121001220 0000220000 2000101121 1211110210 1210101113 1003310102 011000021? ????110111 1010100001 120112?111 1010100111 1

***Dymecodon pilirostris***

1000011010 0010031100 1110200120 0100001101 1112101102 0110002010 1220000220 0110120?00 2000101111 121101??10 121??0111? ?????????? 11?0000110 120?110??? 1010020001 0211120012 11101??01? ?

***Neurotrichus gibbsii***

0001111011 1110131210 0001210021 0100010011 1101101101 0110102021 1121001220 01?1?21010 2000111211 2211010211 1210001113 1103300102 0110000210 0001210121 1111000001 1200120011 1011110112 0

***Scapanulus oweni***

?????00010 1010133311 1100210120 0110001011 1112011002 011010102? 121??02020 0000?22220 2110101210 201111?211 0211112113 11?????113 010?0?0310 ???121?2?? ?????????? ?????????? ??1??????? ?

***Parascalops breweri***

0000000110 0000033311 1100210120 0100001001 0121111101 0110101021 1120?02020 0000222220 2110101210 3011110311 0211112113 1113411113 0100010310 1101210231 1111000011 1110120111 101111?20? ?

***Scalopus aquaticus***

0001100112 0011133310 1101211120 0110001011 0121101002 0110012020 1221010120 0000223121 2010101210 3011111311 0211112113 1113411113 1100000311 2200110231 1111010011 1011120111 1011100212 1

***Scapanus***

0000000112 0001133311 1100210110 0100001011 1121001001 0110011020 1221011120 000?223120 2010101220 3011111311 0211112113 1113311113 0100000311 2100110231 1110010001 0200020011 101111?211 0

***Condylura cristata***

0000000010 0000031100 1000211020 0100001001 0011111120 0110102021 1200?11120 1101122010 2000111231 2010110311 1210102112 1113310113 0110001210 1101210131 1111000001 0201120101 1010111112 2
